# Long Non-Coding RNAs in Immune Thrombocytopenia: A Narrative Review of Pathogenesis and Potential Clinical Implications

**DOI:** 10.3390/cells15212018

**Published:** 2026-11-05

**Authors:** Wojciech Homenda, Dominika Strzała, Aleksandra Połom, Rafał Moraczyński, Krzysztof Homenda, Sylwia Paszek, Natalia Potocka-Wojtowicz, Izabela Zawlik

**Affiliations:** 1 Department of Nursing and Emergency Medicine, Pomeranian University, 76-200 Słupsk, Poland; 2 Department of Hematology and Bone Marrow Transplantation, Provincial Specialist Hospital, 76-200 Słupsk, Poland; dominika.strzala2000@gmail.com (D.S.); aleksandra.polom12@gmail.com (A.P.); r.moraczynski@szpital.slupsk.pl (R.M.); krzyhom@outlook.com (K.H.); 3 Doctoral School, Faculty of Health Sciences, Pomeranian University, 76-200 Słupsk, Poland; 4 Department of General Genetics, Faculty of Medicine, Collegium Medicum, University of Rzeszow, 35-959 Rzeszow, Poland; spaszek@ur.edu.pl (S.P.); npotocka@ur.edu.pl (N.P.-W.); izawlik@ur.edu.pl (I.Z.); 5 Laboratory of Molecular Biology, Centre for Innovative Research in Medical and Natural Sciences, Collegium Medicum, University of Rzeszow, 35-959 Rzeszow, Poland

**Keywords:** long non-coding RNAs, immune thrombocytopenia, gene regulation, targeted therapies, diagnostic biomarkers, autoimmunity, T cells, B cells, platelets

## Abstract

**Highlights:**

**What are the main findings?**

- Altered lncRNA expression in immune thrombocytopenia is linked to immune dysregulation, bleeding severity, and treatment response.
- Findings vary across studies, indicating that the roles of individual lncRNAs are not fully understood.

**What are the implications of the main findings?**

- LncRNAs are potential biomarkers and therapeutic targets that may help guide individualized management of immune thrombocytopenia.
- Clinical validation and standardized measurement methods are needed before these findings can inform routine care.

**Abstract:**

**Background/Objectives:** Immune thrombocytopenia (ITP) involves immune-mediated platelet destruction and impaired platelet production. Long non-coding RNAs (lncRNAs) regulate immune responses, but their clinical relevance in ITP remains uncertain. This narrative review examines disease-associated expression and mechanistic findings, distinguishing translational potential from established clinical utility. **Methods**: English-language literature identified through PubMed, Scopus, and Google Scholar was synthesized narratively. Eight named lncRNAs were prioritized because the cited ITP studies provided transcript-specific patient data or functional evidence; exploratory expression profiling and evidence from other diseases were considered separately. Study design, sample size, specimen type, treatment exposure, and validation status informed interpretation. **Results**: The selected transcripts were *HCP5*, *NEAT1*, *lnc-DC*, *IFNG-AS1*, *MEG3*, *GAS5*, *MALAT1*, and *THRIL*. Among the clinical studies with reported sample sizes, ITP groups generally included 20–90 patients, except for a *GAS5* polymorphism study involving 302 patients. An independent prognostic association for low *HCP5* expression was reported in a single study of 40 patients and has not been externally validated. Other studies described exploratory associations with bleeding, disease duration, or treatment response. Distinguishing primary from secondary ITP and selecting patients for splenectomy are not established applications. Cell experiments and a mouse model support mechanistic hypotheses but do not demonstrate therapeutic efficacy in humans. **Conclusions**: The evidence supports biological plausibility and translational investigation, rather than routine diagnostic, prognostic, or treatment-guiding use. Prospective validation in clinically representative cohorts and standardized assays are required.

## 1. Introduction

Immune thrombocytopenia (ITP) is an autoimmune disorder characterized by increased reticuloendothelial clearance of platelets and insufficient compensatory platelet production. It affects both children and adults and frequently follows a chronic course in adults. The reported annual incidence in adults is approximately 3.3 cases per 100,000 individuals [1]. Despite advances in understanding the underlying mechanisms of ITP and the availability of established treatment approaches, uncertainties remain regarding its pathogenesis, assessment of disease severity, and treatment selection.

Long non-coding RNAs (lncRNAs) have attracted interest as potential regulators of autoimmune processes. These transcripts are generally longer than 200 nucleotides and have limited protein-coding potential, although some can encode small peptides with biological functions [2]. LncRNAs are implicated in cancer, cardiovascular disorders, and neurological diseases. They also regulate cellular and humoral immune responses, supporting investigation of their contribution to autoimmune disorders such as ITP [3,4].

Studies of lncRNA expression in ITP suggest that these transcripts may provide insights into disease pathogenesis and serve as diagnostic and prognostic biomarkers. Their possible associations with treatment responsiveness have also prompted interest in their potential to inform therapeutic decisions [5,6]. These applications, however, require further investigation and clinical validation.

ITP is classified as primary when no underlying cause or associated condition is identified and as secondary when associated with conditions or exposures such as other autoimmune diseases, medications, or viral infections [7]. Diagnosis relies on excluding alternative causes of thrombocytopenia because no definitive diagnostic test is available [8]. The rate of misdiagnosis remains high, although it is difficult to estimate. This diagnostic challenge highlights the need for reliable biomarkers.

This narrative review examines lncRNA expression and function in ITP, focusing on *HCP5*, *NEAT1*, *lnc-DC*, *IFNG-AS1*, *MEG3*, *GAS5*, *MALAT1*, and *THRIL* because the cited ITP literature provides transcript-specific patient observations or functional experiments for these candidates. This focused selection is not an exhaustive inventory of all dysregulated lncRNAs and does not establish that these are the only transcripts with clinical data. The review distinguishes expression associations, mechanistic experiments, and proposed clinical applications, with particular attention to inconsistent findings and the need for independent validation.

## 2. Materials and Methods

This is a focused narrative review, rather than a systematic review or meta-analysis. We searched PubMed, Scopus, and Google Scholar for English-language literature using “immune thrombocytopenia”, “idiopathic thrombocytopenic purpura”, “ITP”, “long non-coding RNA”, “lncRNA”, “autoimmune diseases”, “ITP treatment”, and “targeted therapies”.

The core scope was original research reporting a named lncRNA in clinically characterized ITP samples, its association with disease phenotype or treatment response, or functional perturbation in an ITP-relevant cell or animal model. Studies reporting only high-throughput expression changes or predicted networks were retained as exploratory context, not as equivalent evidence for a validated biomarker or causal mechanism. Reviews and experiments in other diseases were used to explain lncRNA biology and were explicitly distinguished from direct ITP evidence. Duplicate records and reports without an ITP-specific observation or a defined contribution to the mechanistic, diagnostic, or treatment background were outside the focused synthesis. No minimum patient sample size was imposed, and healthy-control comparisons were not treated as proof of diagnostic specificity.

The eight focal transcripts were selected based on the availability of transcript-specific clinical observations or functional ITP experiments, not on the largest fold changes, statistical significance alone, or a presumption of clinical readiness. Li and colleagues reported 1177 upregulated and 632 downregulated lncRNAs in their microarray analysis [6]; most of these signals were discovery-level observations lacking the detailed clinical or mechanistic characterization needed for individual subsections. The same microarray study also reported qRT-PCR confirmation and exploratory discrimination for *ENST00000440492*, *ENST00000528366*, *NR_038920*, and *ENST00000552576* [6]. These additional candidates demonstrate that the eight focal RNAs are not the only lncRNAs with patient-derived ITP data; they were not included in separate mechanistic subsections because the review focuses on the selected transcripts.

For interpretation, patient expression and genetic association studies were distinguished from longitudinal clinical observations, cell perturbation experiments, animal experiments, and computational profiling. Where reported, patient number, age group, specimen, disease phase, treatment exposure, comparator, and independent validation were considered qualitatively; no formal risk-of-bias tool or evidence-grading framework was applied. Small studies were retained for hypothesis generation, with their limitations stated. The year 1994 serves as the conventional cutoff point for the search, as established by the authors: the bibliography includes treatment literature from that year, which does not justify a lower date limit for lncRNA biology, and relevant foundational work, including the original *GAS5* report from 1988, predates it [9]. These reporting limitations constrain reproducibility, as discussed in Section 8 [10].

The diagnostic uncertainty motivating biomarker research is illustrated by the McMaster ITP Registry. Among 295 adults initially classified as having primary ITP, 36 (12.2%) were subsequently reclassified; 10 of 319 initially assigned another diagnosis were later classified as primary ITP. These figures reflect a selected tertiary-care registry and longitudinal reassessment, not a universal diagnostic error rate. A clinically useful lncRNA test should therefore be evaluated against the range of alternative causes encountered in practice, including inherited thrombocytopenia and marrow disorders, as well as healthy controls [11].

## 3. Immune Thrombocytopenia (ITP)

### 3.1. Etiology and Pathogenesis

ITP affects both children and adults, with a higher prevalence in females [12]. Its etiology and clinical course differ between children and adults; in children, ITP often follows a viral infection and resolves spontaneously [13]. The annual incidence in adults is approximately 3.3 cases per 100,000 individuals [1]. Owing to its predominantly chronic course, ITP has a higher prevalence in adults, estimated at 9.5–12.1 cases per 100,000 individuals [14].

ITP is classified according to disease duration as newly diagnosed during the first 3 months after diagnosis, persistent between 3 and 12 months, and chronic beyond 12 months [15]. During the newly diagnosed phase, diagnostic testing focuses on confirming the diagnosis. Historically, clinicians used the term “acute ITP” to describe childhood disease with a short, self-limiting course; adults more frequently develop chronic disease.

The pathogenesis of ITP involves several interacting mechanisms that are not yet fully understood. A central feature is the loss of immune tolerance, leading to the production of autoantibodies against platelets [16]. These antibodies opsonize platelets, facilitating their clearance by the reticuloendothelial system. Antibody-mediated mechanisms and T-cell activity can also promote megakaryocyte apoptosis and impair platelet production [17]. T-cell dysfunction, including abnormalities in cytotoxic T cells, regulatory T cells (Tregs), and the balance between T-helper 1 (Th1) and T-helper 2 (Th2) responses, further contributes to impaired self-tolerance and platelet destruction [18].

Genetic factors may also contribute to susceptibility to ITP. Reported polymorphisms involve Fcγ receptors, chemokines, cytokines, transcription factors, and genes within the major histocompatibility complex (MHC) [19]. Altered gene expression has also been implicated in immune dysregulation [6]. Elevated levels of proinflammatory cytokines, particularly interleukin (IL)-17, IL-2, and interferon-γ (IFN-γ), promote autoantibody formation. Reduced levels of IL-4, IL-10, and transforming growth factor-β (TGF-β) have also been observed. These changes may favor an inflammatory environment that supports autoreactive immune responses.

Dendritic cells (DCs) are the most effective antigen-presenting cells; they process and present antigens to T cells and help regulate immune responses [20]. Dysregulated presentation of self-antigens can contribute to the breakdown of immune tolerance and the development of autoimmune diseases [21]. Viral infections may disrupt these processes and have been associated with the onset of ITP, particularly in children. Influenza has been reported as a potential trigger, and thrombocytopenia has also been described in association with COVID-19 [22,23].

In addition to increased platelet destruction, impaired platelet production contributes to thrombocytopenia in ITP. Bone marrow megakaryocyte numbers are often increased but may be within the normal range. Abnormalities in megakaryocyte maturation can limit effective platelet production [24]. The resulting thrombocytopenia may manifest as mucocutaneous bleeding. The conventional diagnostic threshold for ITP is a platelet count below 100 × 10^9^/L after exclusion of other causes. Bleeding risk increases at very low counts, but no single platelet threshold reliably predicts an individual patient’s bleeding severity [7,25].

### 3.2. Clinical Presentation

ITP is characterized clinically by an increased tendency to bleed, although bleeding does not correlate linearly with platelet count [26]. The clinical course varies, with most children achieving spontaneous resolution within a year [27]. Mortality among patients with ITP is approximately 1.5 times higher than that in the general population and varies with disease severity. Excess mortality is not confined to bleeding: infection, other comorbidities, and treatment-related complications also contribute. Mortality estimates depend on population, age, disease phase, and follow-up [28].

### 3.3. Treatment

Treatment aims to prevent clinically significant bleeding by maintaining a platelet count sufficient for adequate hemostasis while minimizing adverse effects. The target platelet count depends on age, bleeding, comorbidities, medications and planned procedures; a response threshold should not be equated with remission or normal hemostasis [7,8].

Patients with ITP undergoing surgery usually require treatment to increase their platelet count immediately before the procedure. The choice and timing of treatment depend on the baseline platelet count, the bleeding risk associated with the procedure, and its urgency. Treatment options include intravenous immunoglobulin and platelet transfusion.

Treatment guidelines for ITP are based on consensus informed by many years of clinical observation, with bleeding severity and platelet count guiding treatment decisions. Treatment is indicated for patients with active bleeding, regardless of platelet count, as well as those with platelet counts below 10 × 10^9^/L and most patients with counts between 10 and 15 × 10^9^/L [7,8].

#### 3.3.1. First-Line Treatment

Glucocorticoids are the most commonly used first-line treatment for ITP. Regimens include dexamethasone at 40 mg/day for 4 days or prednisone at 1 mg/kg of body weight, with the prednisone dose tapered once the platelet count increases [29]. This treatment aims to reduce autoantibody-mediated platelet sequestration through Fcγ receptors [30]. Changes in Treg numbers have been reported with rituximab-based treatment [31]. In one study, low-dose rituximab combined with short-term glucocorticoids increased Treg levels; this combination study should not be treated as evidence for glucocorticoids alone [32].

Intravenous immunoglobulin (IVIg) is used when a rapid increase in platelet count is necessary, such as in patients with extremely low platelet counts or bleeding manifestations. It is administered at 1 g/kg of body weight for 1–2 days and is considered the safest therapy for ITP during pregnancy [7,33]. Its mechanisms of action are not fully understood. Proposed mechanisms include blockade of macrophage Fc receptors to reduce antibody-mediated platelet clearance and receptor saturation to promote autoantibody clearance. IVIg also affects dendritic cells and modulates T-cell responses, increasing Tregs and reducing Th17 cells [34,35]. Additional effects include inhibition of pro-inflammatory cytokine production, such as IFN-γ, and the suppression of complement activation [36,37].

Polyclonal anti-D immunoglobulin may also be considered as first-line treatment in Rh-positive patients with an intact spleen. It offers an alternative to IVIg when a rapid, but often temporary, increase in platelet count is required. Its use is limited, partly by availability [7], and carries a risk of hemolysis. Like IVIg, its mechanisms of action remain incompletely understood. Effects on B lymphocytes, including reduced autoantibody production, have been reported [38].

Responses to first-line treatment are often transient. Approximately 50% of patients treated with dexamethasone or prednisone maintained a response at 6 months [39]. IVIg produces the most rapid increase in platelet count, usually within 1–3 days. Response rates of up to 85% have been reported, although the response is generally short-lived [40].

#### 3.3.2. Treatment of Refractory/Relapsing ITP

Historically, splenectomy is the most effective ITP treatment, targeting the primary site of antiplatelet antibody production [41]. Due to surgical, thrombotic, and infectious risks necessitating vaccination, it is typically reserved for patients failing drug therapy at least 12 months post-diagnosis [8]. While ~60% of patients maintain normal platelet counts at 5 years [42], ~20% fail or experience early recurrence [7].

Rituximab, an anti-CD20 monoclonal antibody, reduces B-cell numbers and autoantibody production while improving T-cell function [43,44,45]. Approximately 50% of refractory ITP patients respond, but effects are often transient, with only ~30% maintaining a response at 6 months [46,47].

Thrombopoietin receptor agonists (TPO-RAs)—oral eltrombopag/avatrombopag and subcutaneous romiplostim—stimulate platelet production and may improve Treg activity [7,48,49]. While some patients achieve treatment-free remission, responses vary. Risks include thrombosis, marrow reticulin, and liver-test abnormalities with eltrombopag [7,48].

Fostamatinib is an oral Syk inhibitor. In phase 3 trials, 18% achieved a stable response (≥50 × 10^9^/L) with a median time of 15 days; common adverse events included diarrhea, hypertension, nausea, and increased ALT [50]. Conversely, a 2024 Spanish observational study of 138 adults reported a 79% response rate (≥30 × 10^9^/L) in a median of 11 days [15,51]. The drug was well-tolerated, with Grade 3 or 4 adverse events occurring in 9.4% of the cohort [51].

Rilzabrutinib, an oral BTK inhibitor, limits macrophage-mediated platelet destruction and autoantibody production. In its phase 3 trial for persistent/chronic ITP, 64% of patients responded within 12 weeks (median 36 days), though it does not establish a perioperative bridging indication [52].

## 4. Long Non-Coding RNA (lncRNA)

Encompassing a heterogeneous group of transcripts, long non-coding RNAs (lncRNAs) are conventionally defined by a length of >200 nucleotides and a purported lack of protein-coding potential. Nevertheless, this dimensional cutoff remains a procedural artifact rather than a definitive biological boundary, evidenced by annotated lncRNAs containing short open reading frames that yield bioactive micropeptides [2,53,54].

LncRNAs participate in gene regulation, including post-transcriptional regulation, and play important roles in cellular and humoral immune responses [3,4]. They contribute to numerous physiological processes and are implicated in cancer, cardiovascular disorders, neurological disease, and autoimmune disorders. In both innate and adaptive immunity, lncRNAs regulate gene expression and influence immune-cell function and homeostasis [3,4,55,56].

Advances in transcript identification have expanded the catalog of human lncRNAs. LncBook 2.0 reported approximately 95,243 lncRNA genes and 323,950 transcripts [57]. The LncRNADisease v3.0 database contains 25,440 experimentally supported disease-association entries covering 6066 lncRNAs, 10,732 circular RNAs and 566 diseases [58].

Most studied lncRNAs are transcribed by RNA polymerase II and frequently undergo 5′ capping, splicing and polyadenylation, but exceptions include intron-derived and circular species and transcripts with noncanonical 3′ ends. Their abundance, exon structure, subcellular distribution and stability vary considerably. Compared with protein-coding mRNAs, lncRNAs are generally less stable and expressed at lower levels, often in a cell- or tissue-specific manner [59,60]. *MALAT1* is a prominent example of a highly abundant nuclear lncRNA rather than a typical low-abundance transcript [53,54,61].

LncRNAs may overlap protein-coding genes in the sense or antisense orientation, arise from introns, lie between genes or be transcribed divergently from a promoter. These genomic categories describe location and orientation, not mechanism. Nuclear retention can favor interactions with chromatin, nascent RNA and nuclear proteins, whereas cytoplasmic localization permits interactions with messenger RNAs, ribosomes, miRNAs and signaling proteins. Isoform identity, RNA folding and expression in particular cell states can determine function. Sequence conservation is often lower than in protein-coding genes, although promoter activity, splice sites, short motifs, structures or synteny may be conserved; lack of primary-sequence conservation alone does not establish lack of function [53,54,62].

Three additional properties help explain why lncRNAs cannot be treated as a single mechanistic class. First, a locus may generate several isoforms with different interaction domains or subcellular distributions. Second, the effective concentration of an RNA at its transcription site may be high even when its average cellular abundance is low. Third, RNA folding can expose or conceal protein- and nucleic-acid-binding motifs. Consequently, measurements of total transcript abundance do not by themselves identify the active isoform, establish target engagement or predict the magnitude of a regulatory effect [53,54,61].

The distinction between an RNA molecule and its genomic locus is especially important. In a genetic dissection of 12 lncRNA-producing loci in mouse cells, five influenced a neighboring gene, but the observed local effects did not require the specific mature lncRNA sequences. Promoter activity, transcription and splicing instead accounted for these effects. Such findings do not imply that mature lncRNAs are generally dispensable; they show why deleting a locus and depleting its RNA can produce different outcomes and must be interpreted separately [63].

### 4.1. Classification of lncRNAs

**Figure 1:**
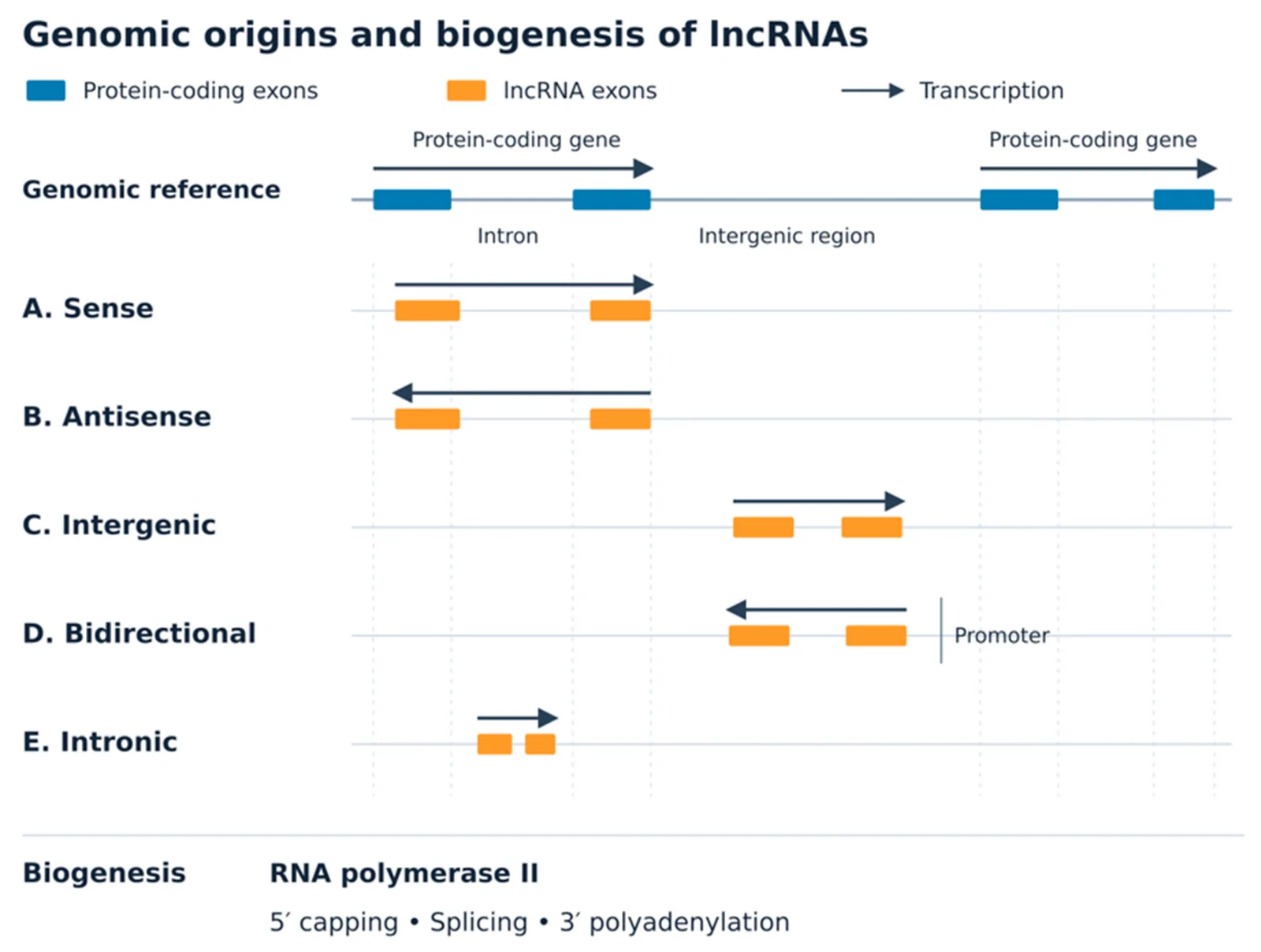
Genomic origins and biogenesis of lncRNAs. The diagram illustrates common genomic arrangements and canonical RNA polymerase II-dependent processing; 5′ capping, splicing and 3′ polyadenylation are not universal requirements for all lncRNAs. Arrows indicate transcriptional direction; blue and orange bands denote protein-coding exons and lncRNA exons, respectively. Intron-derived circular RNAs and transcripts with noncanonical 3′ ends are discussed in the text. Conceptual content adapted from reference [60].

LncRNAs are a highly diverse class of transcripts with distinct molecular mechanisms and functions. Their low sequence conservation complicates sequence-based classification, prompting the development of alternative approaches based on genomic context and molecular characteristics [53]. A widely used approach classifies lncRNAs according to their genomic location relative to protein-coding genes. These categories include intronic, intergenic, antisense, bidirectional, and sense RNAs (Figure 1) [62].

Genomic, structural and functional classifications are complementary. The genomic categories in Figure 1 specify where a transcript originates relative to a selected protein-coding annotation; linear versus circular structure, nuclear versus cytoplasmic localization, and cis versus trans activity describe different properties. These categories may overlap: a divergent transcript can also be antisense to a more distant gene, and an intronic transcript may be classified differently when an alternative host-gene isoform is considered. Classification should therefore report the reference annotation, strand and transcript boundaries, rather than infer function from the gene name alone [54,62,64].

### 4.2. Antisense lncRNAs

Antisense RNAs are transcribed from the DNA strand opposite that of a protein-coding gene and often overlap its intronic or exonic regions. They can influence gene expression through transcriptional interference, chromatin remodeling, and RNA–RNA duplex formation. Their dysregulation contributes to the development of cardiovascular diseases, cancers, and neurological disorders [65,66].

Natural antisense transcripts may overlap a coding gene at its 5′ end, 3′ end or internal exons, creating different opportunities for regulation. Complementary RNA regions can influence RNA stability, processing or translation, whereas convergent or overlapping transcription can alter polymerase access and local chromatin. In the *BACE1* system, *BACE1-AS* increased the stability of the sense mRNA and supported a feed-forward increase in β-secretase expression under cellular stress. This is a demonstrated post-transcriptional mechanism in an Alzheimer’s disease-related system, rather than evidence for the same pathway in ITP [65,67].

Antisense activity can also increase translation without increasing the corresponding mRNA. In mouse cells, anti-sense *Uchl1* required an overlapping recognition region and an inverted SINEB2 element to enhance UCHL1 synthesis; mTORC1 inhibition promoted its cytoplasmic redistribution and association of the sense mRNA with active polysomes. Conversely, *Airn*-mediated silencing of *Igf2r* depended on transcription across the *Igf2r* promoter rather than on the mature *Airn* product. Together, these examples show that antisense orientation can support activating or repressive effects through distinct mechanisms [68,69].

### 4.3. Intron-Derived lncRNAs

Intron-derived lncRNAs originate within intronic regions of protein-coding genes, either through independent transcription or through processing of host-gene precursor RNA. Their production must be distinguished from ordinary intron retention or incompletely processed pre-mRNA. A linear intronic transcript, an excised lariat and a circular intronic RNA have different termini, processing requirements and potential interaction partners; intronic origin does not identify a single biochemical pathway [54,64,70].

Circular intronic RNAs can form when particular excised introns escape normal debranching. Zhang and colleagues identified sequence features near the branchpoint and 5′ splice site that favored their accumulation; selected circular intronic RNAs were enriched in the nucleus and associated with regulation of their parental genes. These molecules should be distinguished from exonic circRNAs generated by back-splicing. Their inclusion in an lncRNA discussion reflects length and noncoding properties, while their circular topology warrants separate experimental identification [70].

*SPRY4-IT1* illustrates a different intronic transcript. It was initially characterized as an intron-derived RNA enriched in melanoma cells and predominantly localized in the cytoplasm; depletion impaired cell growth and invasion and increased apoptosis in those models. Thus, an intronic origin does not require nuclear localization or action on the host gene. These observations provide a mechanistic comparator for lncRNA research but do not establish *SPRY4-IT1* expression changes or a pathogenic role in ITP [71].

### 4.4. Sense lncRNAs

Sense lncRNAs are transcribed in the same orientation as an overlapping protein-coding gene. In commonly used genomic classifications, sense-overlapping transcripts share part of the coding locus, whereas sense-intronic transcripts lie within its introns without overlapping annotated coding exons. These definitions concern orientation and transcript boundaries, not whether a transcript is translated. Classification can vary between annotation systems and should therefore be stated explicitly [64].

A major analytical challenge is separating a genuine sense lncRNA from an alternative coding-gene isoform, read-through transcript or incompletely processed precursor. Strand-specific sequencing, mapping of transcription initiation and termination, and validation of transcript-specific splice junctions can clarify its identity. Where sequences are shared, an assay directed only against a common exon may measure both coding and noncoding isoforms. Similarly, RNA depletion may affect the overlapping mRNA and create an apparent lncRNA phenotype. Functional attribution therefore requires isoform-specific perturbation and measurement of the neighboring coding transcript and protein [54,63,64].

### 4.5. Bidirectional lncRNAs

Bidirectional or divergent lncRNAs initiate near a protein-coding promoter and are transcribed in the opposite direction. Definitions often use a short distance between transcription start sites, but the exact cutoff depends on the annotation framework. A shared accessible promoter region can explain correlated expression of the coding and noncoding transcripts. Divergent transcription does not, by itself, demonstrate enhancer activity or prove that the mature RNA regulates its coding partner [54,60,64].

The upperhand–*Hand2* locus provides an informative example. In mice, upperhand initiates approximately 150 base pairs upstream of *Hand2* and shares a bidirectional promoter with it. Premature termination of upperhand transcription disrupted the active enhancer environment and *Hand2* expression, producing severe cardiac developmental defects. In contrast, depletion of the mature upperhand RNA did not reproduce the effect on *Hand2*. This establishes a role for transcription through the locus in that system and cautions against interpreting co-expression as evidence for an RNA-mediated trans mechanism [72].

### 4.6. Intergenic lncRNAs

Intergenic lncRNAs originate in the genomic intervals separating protein-coding genes and do not overlap annotated protein-coding loci [73]. They regulate gene expression by modulating RNA stability and influencing chromatin-modifying complexes. Their tissue-specific expression makes them potential biomarkers for guiding targeted therapy [53].

Long intergenic noncoding RNAs (lincRNAs) are often useful for functional investigation because they lack annotated overlap with coding genes, although their promoters can still function as regulatory DNA elements. Their genomic separation from coding loci does not imply action in trans: an intergenic RNA can influence a nearby gene in cis or associate with distant targets through chromosomal contacts. *HOTTIP*, for example, was associated with WDR5/MLL complexes and promoted an active H3K4-trimethylated state across the 5′ *HOXA* region in developmental models. Chromosomal proximity helped determine its targets [63,73,74].

Intergenic transcripts also regulate immune-cell responses. In mouse myeloid cells, *lincRNA-Cox2* was induced after Toll-like receptor stimulation and contributed to both activation and repression of inflammatory genes; repression involved hnRNP A/B and A2/B1. *Morrbid* regulated the pro-apoptotic neighboring gene *Bcl2l11* and the survival of short-lived myeloid cells, including neutrophils, eosinophils and classical monocytes. These examples broaden the functional context beyond miRNA competition, while disease-specific evidence remains necessary before assigning either pathway a role in ITP [55,56].

### 4.7. How lncRNAs Regulate Gene Expression

The functional unit may be the mature RNA, its act of transcription, splicing, or a regulatory element at the locus. A transcript may act in cis near its site of synthesis or in trans at distant loci. Nuclear lncRNAs can associate with chromatin regulators, influence recruitment or retention at genomic regions, affect local transcription, alter RNA polymerase activity, and bind nascent RNA or splicing factors. Cytoplasmic lncRNAs can influence mRNA stability, translation and protein localization or modify signal transduction through protein interactions. These mechanisms are context-dependent, and a physical association alone does not demonstrate that recruitment is direct or causally necessary [53,54,61].

At the molecular level, an RNA can recognize another RNA through sequence complementarity, associate with a protein through a structured or short sequence motif, or contact chromatin directly or through an intermediary. RNA–DNA triplex formation offers one experimentally investigated targeting mechanism: *MEG3* associated with distal regulatory regions of TGF-β pathway genes through triplex-forming interactions in breast cancer cell models. This differs from an RNA–DNA hybrid and should not be assumed for every chromatin-associated lncRNA or for *MEG3* in ITP without direct testing [75].

Regulation can also occur after a protein has been synthesized. *GAS5*-associated effects on STAT3 ubiquitination in ITP models illustrate control of protein turnover, whereas *lnc-DC*-dependent protection of STAT3 phosphorylation illustrates modulation of signaling activity. The shared involvement of STAT3 does not imply an identical mechanism: protein abundance, phosphorylation, binding partners and cell lineage are separate determinants of the final response. This distinction helps interpret why different lncRNAs can influence the same inflammatory pathway in different directions [76,77].

Some transcripts contain miRNA-binding sites and have been proposed to function as competing endogenous RNAs (ceRNAs). A credible ceRNA mechanism requires sufficient relative abundance, co-localization, accessible binding sites and evidence that altering the proposed interaction changes the target pathway at physiological concentrations. Computational prediction, a luciferase reporter or correlation alone is insufficient. In immune thrombocytopenia, apparent transcript differences may reflect treatment exposure or altered immune-cell proportions; functional experiments in defined cell populations are needed to establish causality [61,78,79].

Quantitative experiments reinforce these requirements. Denzler and colleagues found that substantial increases in competing target-site abundance were needed to relieve miRNA-mediated repression in a hepatocyte miR-122 system. The measured threshold is specific to that experimental setting and is not a universal cutoff, but it demonstrates why the number of accessible binding sites matters. Accordingly, an ITP ceRNA model should measure endogenous RNA abundance and localization and test whether disrupting the proposed binding site changes the phenotype without requiring nonphysiological overexpression [80].

### 4.8. Four Molecular Archetypes: Signal, Decoy, Guide and Scaffold

Signal: lncRNA expression can integrate a particular developmental stage, inflammatory stimulus or combination of transcription factors and thereby mark an underlying regulatory state. A signal transcript may itself be functional or simply report activation of its locus; expression alone cannot distinguish these possibilities. *IFNG-AS1* illustrates stimulus- and lineage-associated transcription at an immune locus, although its effects extend beyond serving as a signal [61,81,82].

The signal category is most informative when the inducing stimulus, responsive cell population and timing are defined. An *IFNG-AS1* increase in activated T cells may convey different information from a serum increase in a mixed patient cohort. In the *Ifng-as1* locus, experimental work showed contributions from both noncoding transcription and local DNA architecture to the magnitude of IFN-γ responses. Thus, a signal may identify an activated locus while the locus itself also participates in feedback control; expression measurements alone cannot separate these roles [82].

Decoy: an RNA reduces the availability of a regulatory molecule by binding it. A protein decoy need not alter the total amount of that protein; it may instead compete with its normal DNA or RNA targets. *GAS5* provides a well-characterized example: a structured RNA element mimics a glucocorticoid response element and binds the DNA-binding domain of the glucocorticoid receptor, limiting receptor engagement with responsive genes in growth-arrested or nutrient-deprived cells. This mechanism was established outside ITP and should be distinguished from the *GAS5*–STAT3 pathway investigated in ITP models [61,76,83].

Guide: an RNA helps position or retain regulatory proteins at particular genomic sites. *XIST* acts predominantly in cis on the X chromosome from which it is transcribed; *HOTAIR* has been described as a transacting regulator associated with chromatin complexes, although target specificity and generality remain context-dependent. Distinguishing direct recruitment from secondary effects requires locus-specific perturbation and interaction experiments [53,61].

Guide activity requires both target recognition and a regulatory effector. *XIST* provides chromosome-restricted targeting coupled to several silencing pathways, whereas *HOTTIP* illustrates recruitment associated with gene activation. *HOTAIR* was originally characterized at the *HOXC* locus and linked to repression at the distant *HOXD* locus. These examples show that guide activity can be activating or repressive and cis or trans; the relevant distinction is how a particular RNA–protein system reaches and changes a defined target [74,84].

Scaffold: different RNA regions provide a platform for the assembly of multiple molecular partners or a nuclear structure. *HOTAIR* has been proposed to associate with distinct chromatin-modifying activities; *NEAT1*, particularly its long isoform, is an architectural component of paraspeckles. *MALAT1* interacts with nuclear speckle-associated RNA-processing proteins, but it is not indispensable for speckle formation in mouse knockout models and should not be presented as an obligatory architectural scaffold [61,85,86,87]. The four archetypes overlap: an individual lncRNA can act through more than one mechanism, and neither its name nor subcellular location proves a specific mechanism.

In the original *HOTAIR* scaffold model, distinct RNA regions are associated with PRC2 and the LSD1/CoREST/REST complex, connecting H3K27 methylation with H3K4 demethylation. A scaffold claim therefore implies more than detecting two proteins in an RNA pull-down: the RNA regions involved and their contribution to complex assembly or target regulation should be tested. *NEAT1_2* represents an architectural example in which the long isoform supports paraspeckle construction, whereas *MALAT1* illustrates an association with a nuclear compartment without an absolute requirement for that compartment to form [87,88,89].

### 4.9. XIST and X-Chromosome Inactivation

X-inactive specific transcript (*XIST*; *Xist* in mouse) exemplifies chromosome-scale cis regulation. During early mammalian development, *XIST* is expressed from the future inactive X chromosome and spreads across that chromosome, forming a nuclear RNA compartment. *XIST* repeat domains recruit distinct RNA-binding proteins; the A-repeat associates with SPEN, which engages corepressor machinery, including NCoR/SMRT and HDAC3, to promote histone deacetylation and transcriptional silencing. Other *XIST*-associated pathways recruit Polycomb activities, contributing to repressive chromatin marks, and additional factors such as SMCHD1 support establishment or maintenance in a differentiation-dependent manner [90,91].

Two experimentally separable branches explain how *XIST* couples RNA recognition to chromatin changes. SPEN binds the A-repeat and uses its SPOC effector domain to connect *Xist* to transcriptional corepressors, remodeling machinery and histone deacetylation. In parallel, hnRNPK recognizes a region encompassing the B-repeat and links *Xist* to PCGF3/5-containing PRC1. This promotes H2AK119 monoubiquitination and subsequent Polycomb accumulation, including PRC2-associated H3K27 trimethylation. *XIST* therefore coordinates complementary transcriptional and chromatin pathways rather than acting solely through a direct RNA–PRC2 interaction [92,93].

The inactive chromosome acquires further stable epigenetic features, including DNA methylation at many promoters, while a subset of genes escapes inactivation. Mechanisms and timing differ among cell types, developmental stages and species; *XIST* coating does not imply identical silencing of every X-linked gene. *XIST* is therefore best understood as a cis-acting organizer of several complementary silencing pathways, not a single universal switch. Altered X-chromosome dosage or inactivation maintenance has been investigated in autoimmunity, but a causal role of *XIST* dysregulation in ITP requires direct disease-specific evidence [90,91].

The requirements for initiating silencing are not identical to those for maintaining it after differentiation. In mouse experiments, SPEN was essential for efficient initiation in embryos and embryonic stem cells but dispensable for maintenance in neural progenitors, although it continued to affect some escape-gene expression. Such results explain why *XIST* abundance, chromosome coating and transcriptional silencing must be assessed separately. For autoimmune research, the relevant questions concern the affected immune lineage, differentiation state and individual X-linked genes, rather than a global assumption that all X-linked expression changes reflect failure of *XIST* [92].

Evidence connecting *XIST* to autoimmunity extends beyond chromosome dosage. Dou and colleagues induced a non-silencing *Xist* transcript in male mice; *Xist*-associated ribonucleoprotein complexes were accompanied by autoantibody production and, in a susceptible strain, more severe disease in a pristane-induced lupus model. Human autoimmune sera also recognized several *XIST*-associated proteins. These results support a testable immunogenic-complex mechanism but do not establish that *XIST* causes ITP or that this pathway predicts ITP outcomes [94]. A subsequent study mapped antibody-reactive regions of *XIST*-associated proteins and investigated their release during neutrophil extracellular trap formation; these observations also require ITP-specific validation [95].

### 4.10. MALAT1: Nuclear Organization and RNA Processing

*MALAT1* (also called *NEAT2*) is an abundant nuclear-retained transcript enriched in nuclear speckles, domains containing pre-mRNA processing factors. Unlike conventional polyadenylated mRNAs, its primary transcript is cleaved by RNase P at a tRNA-like structure to generate the mature 3′ end; subsequent RNase Z processing produces the smaller *MALAT1*-associated cytoplasmic RNA (mascRNA). A conserved 3′ triple helix protects the mature long transcript against exonucleolytic degradation despite the absence of a conventional poly(A) tail [96,97].

The *MALAT1* locus demonstrates how a single precursor can generate products with different destinations. RNase P cleavage creates the mature *MALAT1* 3′ end and the 5′ end of a small tRNA-like RNA; further processing yields the approximately 61-nucleotide mascRNA, which accumulates in the cytoplasm. The mature long RNA remains nuclear. Its genomically encoded A-rich region participates in a protective triple-helical structure and should not be equated with the conventional poly(A) tail added to most mRNAs. This processing route also means that an assay measuring an internal *MALAT1* segment does not necessarily establish the integrity of the entire mature transcript [96,97].

*MALAT1* associates with serine/arginine-rich splicing regulators and active chromatin, and perturbation studies have linked it to RNA processing and transcriptional control in selected contexts [86,87]. Nuclear speckles are distinct from paraspeckles, whose organization depends on *NEAT1*. Importantly, mouse *Malat1* knockout studies found preserved nuclear speckle organization and viability under laboratory conditions; this limits claims that *MALAT1* is universally essential for speckle formation. Its effects can depend on cell type, assay, stimulation and species [87]. In ITP, reported expression changes remain candidate associations rather than proof that a specific speckle or splicing mechanism causes thrombocytopenia [98].

In cultured human cells, Tripathi and colleagues found that *MALAT1* associated with serine/arginine-rich proteins, influenced their distribution and phosphorylation, and altered alternative splicing of selected endogenous pre-mRNAs after depletion. These findings support a role in regulating the availability or activity of processing factors. Their interpretation should incorporate the preserved speckles and viability observed in mouse knockout studies: a transcript can modulate selected processing events without being essential for nuclear-body assembly in every biological setting [87,99].

For ITP, a mechanistic *MALAT1* study would therefore need to identify the immune-cell population responsible for altered expression and test defined downstream splicing or transcriptional events. Circulating *MALAT1* could derive from released RNA, extracellular vesicles or residual cells; its concentration cannot establish nuclear speckle activity in a particular leukocyte population. Paired measurements in purified cells and circulation, together with localization and perturbation experiments, would distinguish an accessible biomarker from a causal intracellular regulator [86,98,99].

### 4.11. Disease Relevance and Representative lncRNAs

LncRNAs can be implicated in disease through changes in transcription, isoform usage, RNA stability, localization, genetic variation or altered interaction partners. Dysregulation can influence cell differentiation, inflammatory signaling, tolerance, proliferation or tissue injury. Examples include *XIST* in dosage compensation, *HOTAIR* in chromatin-associated gene regulation, *MALAT1* in nuclear RNA processing, *NEAT1* in paraspeckle assembly, and *H19* in development and imprinting. *GAS5*, *MEG3*, *IFNG-AS1*, *lnc-DC* and *THRIL* provide examples of transcripts studied in immune regulation and ITP; *HCP5* is another immune-associated candidate [53,61,76,81,85,100,101,102,103]. These biological examples do not establish diagnostic validity or therapeutic efficacy in ITP.

*H19* illustrates another route from lncRNA expression to biological function. This developmentally regulated, imprinted transcript contains the precursor of miR-675 in its first exon. In placental models, regulated production of miR-675 influenced growth, including regulation of *Igf1r*; the RNA-binding protein HuR controlled processing of the precursor. Thus, a phenotype assigned to an lncRNA locus can partly arise from a smaller RNA generated from it. *H19* is an instructive example of processing-dependent regulation, rather than an established ITP biomarker or treatment target [104].

The same transcript can show different directions of association across tissues, disease stages, treatments and assays. For biomarker development, studies should report patient selection, disease duration and activity, age, infection and concomitant therapy; specimen type, processing time, RNA integrity, isoform and normalization strategy; and prespecified diagnostic comparators, including other causes of thrombocytopenia. Prospective independent validation should assess discrimination, calibration and added value over clinical evaluation rather than relying solely on case–healthy-control comparisons [8,53,98,105].

For therapeutic development, antisense oligonucleotides or other RNA-directed approaches could in principle reduce a pathogenic transcript, whereas restoration or modulation of a protective function poses different delivery challenges. Tissue targeting, off-target effects, immunogenicity and the risk of disturbing normal immune tolerance require evaluation. Dissecting RNA-mediated activity from promoter or transcriptional effects using complementary genetic and RNA-directed perturbations, followed by rescue experiments, is essential before translation. No lncRNA-directed treatment for ITP is established in routine clinical practice [53,54,61].

The therapeutic direction must follow the specific mechanism. Depleting a pathogenic nuclear RNA, restoring an RNA that promotes degradation of an inflammatory protein, and changing transcription through a regulatory locus are not interchangeable interventions. In particular, an antisense oligonucleotide that reduces mature RNA may not reproduce promoter deletion or premature transcription termination. For candidates with multiple functions, including *GAS5*, domain-specific modulation may ultimately be more informative than changing total RNA abundance. These considerations currently define experimental strategies rather than a validated therapeutic approach for ITP [63,72,76,83].

## 5. LncRNAs in Autoimmune Diseases

Autoimmune diseases result from immune responses directed against the body’s own tissues and organs, causing inflammation and tissue damage. A large UK population-based study discussed by Wen and Li estimated that approximately one in ten individuals was affected by one of the autoimmune diseases examined; this estimate should not be generalized directly to global prevalence [106]. Their development and clinical course reflect interactions among immune dysregulation, environmental factors, abnormal antigen presentation, and genetic predisposition. Genetic factors offer opportunities to identify new diagnostic and prognostic markers [107]. LncRNAs play important roles in cellular and humoral immune responses [3]. They contribute to both innate and adaptive immunity by regulating the homeostasis of immune cells [4].

### 5.1. HLA Complex P5 (HCP5)

*HCP5* is a hybrid HLA class I endogenous retroviral gene within the MHC region and has reported associations with immune-mediated diseases [100]. *HCP5* regulates Treg function in allergic rhinitis through the miR-16/ATXN2L axis [108]. Increased *HCP5* expression in rheumatoid arthritis synovial tissue has been associated with immune-cell infiltration [109]. Genetic and epigenetic associations with systemic lupus erythematosus have also been reported; these findings should be distinguished from evidence validating circulating *HCP5* as a disease-activity biomarker [100].

### 5.2. Exosomal Non-Coding RNAs

Exosomes contribute to the regulation and pathogenesis of immune-mediated diseases [110,111]. These extracellular vesicles, measuring approximately 30–150 nm in diameter, transport RNAs, proteins, and lipids between cells. By transferring their cargo, exosomes can influence antigen presentation and activate T cells by stimulating cytokine production. These mechanisms activate inflammatory pathways that contribute to autoimmune disease [110]. Through intercellular communication, exosomes influence both the innate and adaptive immune responses. Their cargo includes non-coding RNAs, such as microRNAs (miRNAs), circRNAs, and lncRNAs [112]. Evidence indicates that exosomal non-coding RNAs contribute to the development of various diseases, including autoimmune disorders, with lncRNAs playing a role in the regulation of autoimmune processes [111].

### 5.3. Examples of Autoimmune Diseases

#### 5.3.1. Systemic Lupus Erythematosus

Systemic lupus erythematosus (SLE) is an autoimmune disease characterized by autoreactive lymphocyte activation, autoantibody production, and immune complex deposition in multiple organs. Immune-complex-mediated nephritis is the most serious complication of SLE [113]. Dysregulated B-cell activation contributes to immune complex formation. Activated exosomes derived from basophils carry the lncRNA *ENST00000537616*, which binds to miR-330-5p and activates KRAS signaling. This process enhances B-cell activation and promotes immune complex deposition [114]. *NEAT1* has been implicated in immune and inflammatory regulation across disease models; the pathways summarized in a broad review should not be assumed to be established specifically in SLE-associated ITP [115].

#### 5.3.2. Psoriasis

Psoriasis is a chronic, recurrent systemic autoimmune disease in which dysregulated immune responses trigger inflammatory processes, resulting in skin lesions and joint inflammation [116]. Exosomes derived from the human skin keratinocyte cell line HaCaT carry the lncRNA *AGAP2-AS1*, which influences Th1- and Th17-cell differentiation and modulates inflammatory responses [117].

#### 5.3.3. Rheumatoid Arthritis

Rheumatoid arthritis is a chronic autoimmune disease characterized primarily by synovial inflammation that leads to cartilage destruction and bone damage. Progressive joint damage can result in permanent disability [118]. Fibroblast-like synoviocytes (FLSs) play a key role in the pathogenesis of rheumatoid arthritis. Their abnormal activation promotes the secretion of pro-inflammatory mediators such as IL-6, IL-15, and CXCL8. This increases fibronectin production and adhesion molecule expression, promoting leukocyte recruitment to the synovial membrane and inflammation [118,119]. The lncRNA *TRAFD1-4:1*, carried in FLS cell exosomes, increases CXCL1 expression, leading to chondrocyte dysfunction and extracellular matrix damage [120]. *NEAT1* also promotes inflammation by enhancing the differentiation of CD4+ T cells into Th17 cells [115].

## 6. Role of lncRNAs in the Pathogenesis of ITP

### 6.1. HCP5

The study of Liu et al. evaluated 40 patients with immune thrombocytopenia (ITP) and 40 healthy controls to investigate the prognostic value of *HCP5* expression [5]. The authors monitored the patients over a one-year treatment period, classifying them into favorable or poor-prognosis groups based on disease recurrence, bleeding complications, and platelet counts. Patients with hemorrhagic complications were classified according to bleeding severity as mild, moderate, or severe. Patients received treatment according to ITP standards of care. First-line therapy included glucocorticoids and immunoglobulins, while TPO-RAs or rituximab were used for refractory or relapsing disease. After 1 year of treatment, patients were classified into favorable and poor-prognosis groups. Criteria for poor prognosis included disease recurrence within 12 months, hemorrhagic complications, and platelet count <100 × 10^9^/L. *HCP5* expression was significantly lower in patients with ITP than in controls (*p* < 0.001) [5]. The authors showed that *HCP5* expression was significantly reduced in ITP patients and reported an independent association between low *HCP5* levels and their composite poor-prognosis outcome in this single 40-patient cohort. This within-cohort multivariable association is not evidence of an externally validated prognostic test. These results are promising but should be considered preliminary because the study population was relatively small.

*HCP5* should also be interpreted in its genomic context within the HLA region, where immune-associated genetic variation and coordinated transcription can complicate causal attribution. An association between *HCP5* abundance and clinical outcome could reflect a direct RNA effect, variation linked to neighboring immune genes, or differences in the cellular composition of the blood sample. The available ITP cohort supports prognostic exploration, whereas direct perturbation in relevant immune-cell subsets is needed to establish which *HCP5*-dependent process, if any, changes platelet-directed immunity. Findings on Treg regulation in allergic rhinitis provide a candidate pathway for investigation rather than confirmation of that pathway in ITP [5,100,108].

### 6.2. NEAT1

*NEAT1* is an architectural lncRNA whose long isoform supports paraspeckle assembly [85,89]. A primary experimental study linked *NEAT1*-dependent SFPQ redistribution to *IL8* induction after immune stimulation [121], whereas a broader review discusses its roles in stress, immune responses, and other disease settings [115]. These distinct sources should not be used interchangeably. A study at Fayoum University Hospital in Egypt included 60 children with ITP, comprising 42 girls and 18 boys, with a median age of 5 years (range, 1–13 years); serum *NEAT1* expression was elevated relative to healthy controls [101]. This association does not establish a causal *NEAT1* pathway in ITP. Virus-induced *NEAT1* responses in experimental systems provide a mechanistic hypothesis, but a role in HIV-1- or SARS-CoV-2-associated ITP has not been demonstrated by the cited pediatric study [101,115,121].

*NEAT1* has two major isoforms generated by alternative 3′-end processing: the short, polyadenylated *NEAT1_1* and the much longer, noncanonically processed *NEAT1_2*. The long isoform is required for normal de novo paraspeckle assembly and associates with proteins including NONO and SFPQ. HNRNPK can shift processing away from the proximal polyadenylation site, favoring *NEAT1_2* production. Consequently, a total-*NEAT1* assay that amplifies the region shared by both isoforms cannot identify whether a clinical association involves increased paraspeckle-forming capacity [89].

**Figure 2:**
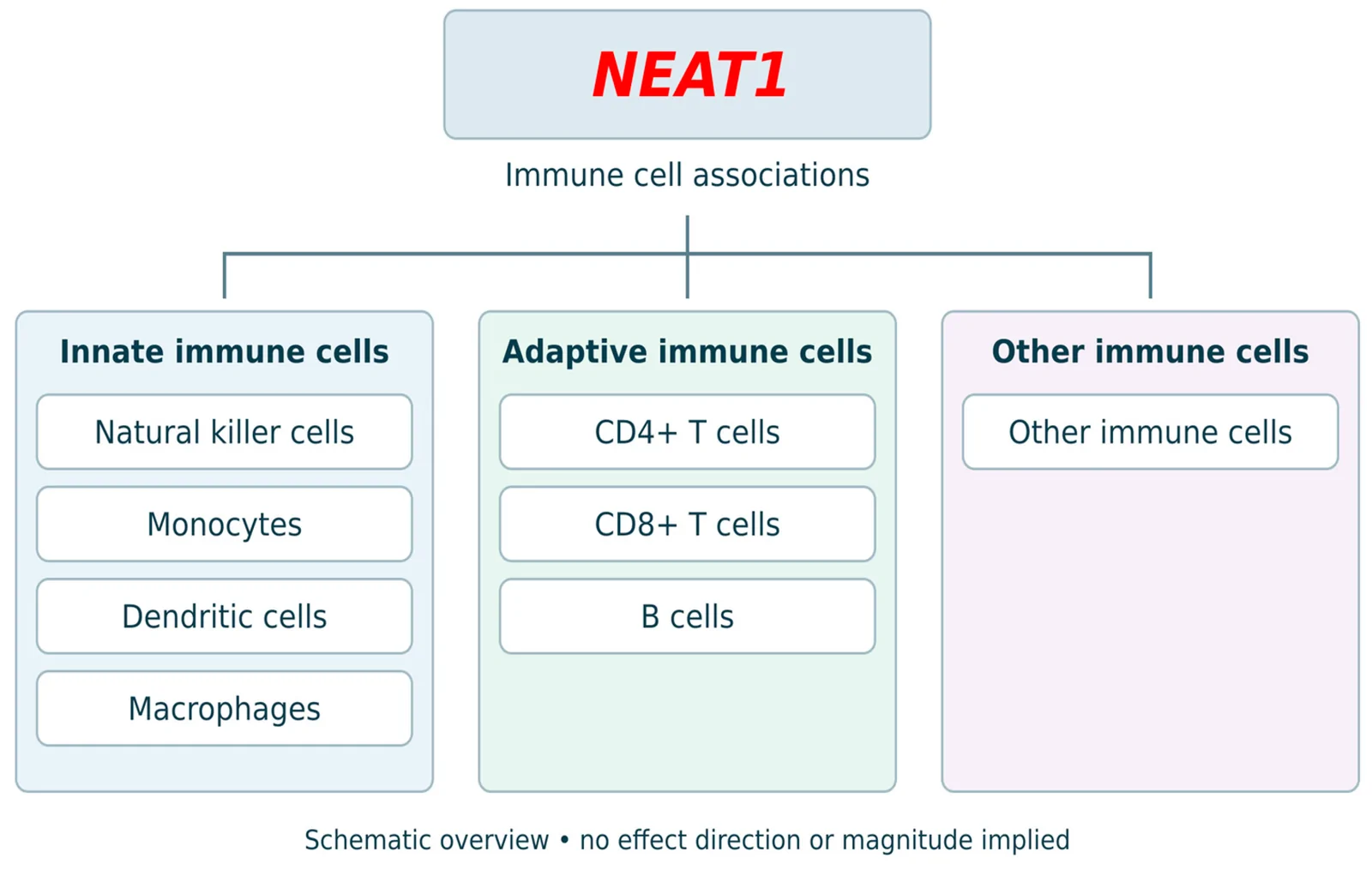
Schematic overview of immune cell populations associated with *NEAT1*, reorganized into innate, adaptive, and other immune cell categories. Conceptual content adapted from reference [115].

A defined immune mechanism was demonstrated in virus-infected or poly(I:C)-stimulated cells: *NEAT1* induction promoted redistribution of SFPQ from the *IL8* promoter into paraspeckles, relieving transcriptional repression. This provides a plausible link between nuclear organization and inflammatory gene expression. However, increased *NEAT1* in children with ITP does not establish that SFPQ redistribution or *IL8* regulation mediates their thrombocytopenia. Isoform-specific quantification, cellular localization and direct measurement of the proposed downstream response would be required to test that link [101,121]. A schematic overview of immune cell populations associated with *NEAT1* can be found in Figure 2.

### 6.3. Lnc-DC

Expressed in dendritic cells, *lnc-DC* promotes STAT3 phosphorylation at tyrosine 705, supporting dendritic-cell maturation and differentiation [77]. Increased *lnc-DC* expression promotes monocyte differentiation into dendritic cells and subsequent T-cell activation. Dendritic cells play a significant role in the pathogenesis of ITP by presenting platelet antigens to T cells [122]. Prednisone treatment reduces dendritic cell numbers, which contributes significantly to the increase in platelet count. The study at the Pediatric Department of Fayoum University Hospital in El Fayoum, Egypt, examined *lnc-DC* expression in the same pediatric cohort described above for *NEAT1* [101]. Relative serum *lnc-DC* expression was significantly higher in patients with the non-chronic form of ITP than in controls [101]. These findings are consistent with those of Zhou and colleagues, who reported dendritic-cell activation in patients with this form of ITP [123].

The original human dendritic-cell study identified a post-translational mechanism: *lnc-DC* bound STAT3 and limited its interaction with the phosphatase SHP1, favoring phosphorylation at Tyr705 and dendritic-cell differentiation. This is distinct from demonstrating that the RNA directly presents platelet antigens or that its circulating abundance measures dendritic-cell activity. The higher serum values reported in non-chronic pediatric ITP and lower plasma values in adult primary ITP therefore address different biological questions. Age, treatment, specimen type and the cellular origin of released RNA should be evaluated before interpreting either direction as a universal disease mechanism [77,101,105].

### 6.4. MEG3

*MEG3* has been investigated in cancer and immune regulation, but findings from malignancy-associated expression studies do not establish a causative role in ITP. In the ITP study by Li and colleagues, *MEG3* was increased in purified CD4+ T cells, and experimental perturbation implicated the *MEG3*–miR-125a-5p axis in altered FOXP3 and RORγt expression. Dexamethasone reduced *MEG3* in vitro; this does not establish that *MEG3* mediates clinical glucocorticoid responsiveness [102]. Its chromatin-associated effects on TGF-β pathway genes were investigated separately in breast cancer models and should be distinguished from ITP findings [75].

In 2023, Naguib and colleagues reported a study involving 45 adults with newly diagnosed ITP and 45 healthy controls [124]. Patients were diagnosed in a hospital or outpatient clinic. Exclusion criteria included HCV infection, other autoimmune diseases, thyroid disease, drug-induced thrombocytopenia, liver cirrhosis, cancer, and chronic kidney disease. The mean age of patients with ITP was 34.58 ± 12.43 years, and 84% were women. The mean platelet count increased from 13.77 ± 12.24 × 10^9^/L before treatment to 123.96 ± 91.86 × 10^9^/L after treatment. Ten patients (22.2%) had organ hemorrhage, while 35 patients (77.8%) had mucocutaneous hemorrhage. Platelet transfusions were required in 29 patients (64.4%). Glucocorticoids were used in 44.4% of patients; other treatments included rituximab (22,4% of patients) and splenectomy (13,3% of patients). *MEG3* levels were significantly higher in patients with ITP than in controls (6.79 ± 1.98 vs. 1.04 ± 0.07; *p* < 0.001) and decreased after treatment. Higher levels were associated with severe bleeding and the need for platelet transfusions. Patients with severe bleeding had higher *MEG3* levels than those with mucocutaneous hemorrhage, whereas patients who responded to glucocorticoids had lower levels than the other treated patients [124].

In the *MEG3* study by Li and colleagues, purified peripheral-blood CD4+ cells from 30 adults with ITP and 30 healthy participants were examined. *MEG3* was increased in patient cells; experimental downregulation of *MEG3* or overexpression of miR-125a-5p increased FOXP3 and reduced RORγt expression. Dexamethasone reduced *MEG3* in vitro. These findings support an experimentally tractable *MEG3*–miR-125a-5p relationship, but transcription-factor expression in cultured cells should not be equated automatically with a clinical change in Treg or Th17 function [102]. A separate patient study examined circulating *MEG3* alongside miR-125a-5p, CXCL13 and NF-κB, illustrating that results from whole blood or circulation and purified T cells cannot be assumed to be interchangeable [124].

*MEG3* also has chromatin-associated functions outside ITP. In breast cancer cell models, it contacted distal regulatory DNA through triplex-forming sequences and influenced TGF-β pathway gene expression. This broadens the mechanistic possibilities beyond a single miRNA interaction, but it does not demonstrate triplex-mediated control in patient T cells. In ITP, experiments should distinguish changes in miR-125a-5p abundance, direct RNA association, transcription-factor expression and functional suppression by Tregs. Rescue with interaction-defective *MEG3* constructs could help determine which molecular feature is necessary for the observed phenotype [75,102].

### 6.5. GAS5

*GAS5* is a multi-exon lncRNA gene at chromosome 1q25 with multiple transcript isoforms. Its name reflects its original identification among growth-arrest-associated transcripts, rather than a uniform effect across diseases [9]. Initially identified for its growth-inhibitory role in several cancers, *GAS5* also participates in the regulation of autoimmune diseases [125]. *GAS5* accumulation is linked to mTOR (mammalian target of rapamycin) inhibition. In human T cells, *GAS5* is required for the antiproliferative effects of mTOR antagonists [126]. Its effects on Th17 differentiation in ITP were investigated separately [76].

Given its role in T-cell regulation, *GAS5* may also contribute to ITP pathogenesis. The clinical significance of promoter polymorphisms, rather than acquired mutations, requires independent validation. Between June 2023 and June 2024, researchers at Quzhou People’s Hospital in China investigated the association between *GAS5* polymorphisms and susceptibility to ITP [127]. The study included 302 adults with ITP (157 men and 145 women) and 300 healthy controls. The mean age was 33 years in both groups. Patients met the diagnostic criteria for ITP, and those with potential secondary causes of thrombocytopenia were excluded. The deletion (D) allele was significantly less frequent in patients with ITP than in controls (23.0% vs. 31.17%) and was associated with a significantly lower risk of developing ITP. The combined frequency of the ID and DD genotypes was also significantly lower in patients than in controls (40.7% vs. 53.3%). Serum *GAS5* levels were significantly reduced in the ITP group [127].

Li and colleagues studied 20 adults with ITP and 20 matched controls as well as an ITP mouse model. *GAS5* expression was reduced in patient peripheral-blood mononuclear cells and mouse spleen. RNA pull-down and immunoprecipitation supported *GAS5*–STAT3 association; in the experimental system, *GAS5* promoted TRAF6-associated STAT3 ubiquitination and reduced Th17 differentiation. *GAS5* overexpression ameliorated disease in the mouse model, but this is preclinical evidence rather than proof of benefit from *GAS5* therapy in humans. Notably, overexpression did not alter Treg differentiation in the reported naïve CD4+ cell experiment [76].

*GAS5* requires particular care because its reported molecular activities can have different implications for treatment. Promoting STAT3 turnover may reduce Th17 differentiation in an experimental ITP model, whereas acting as a glucocorticoid-receptor decoy may attenuate selected glucocorticoid-responsive transcriptional programs in another cellular setting. The latter finding does not establish clinical steroid resistance in ITP. Distinguishing these activities requires measurements of the relevant *GAS5* isoform or domain, receptor-dependent transcription and STAT3 signaling in the same cell population, together with documentation of glucocorticoid exposure [76,83].

### 6.6. MALAT1

*MALAT1*, also known as *NEAT2*, is an abundant nuclear lncRNA of approximately 8 kb encoded at chromosome 11q13.1. It was originally identified through an association with metastasis and survival in early-stage non-small-cell lung cancer; that observational finding did not itself establish that *MALAT1* causes metastasis [128]. *MALAT1* has also been investigated in autoimmune diseases [119,129].

A nearly 2-year study at the Pediatric Clinic of Fayoum University Hospital in Egypt from July 2017 to May 2019 investigated 90 patients younger than 18 years with ITP and an age-matched control group [98]. Patients had platelet counts below 100 × 10^9^/L, and secondary causes of thrombocytopenia were excluded. Sixty patients (66.6%) had newly diagnosed ITP, while the remaining patients had chronic disease. *MALAT1* expression was significantly higher in patients with ITP than in controls and correlated positively with lymphocyte count. *MALAT1* RNA localizes to hundreds of genomic sites in human cells [86]. Yang and colleagues reported that increased *MALAT1* expression may induce SIRT1 expression, which participates in innate immune responses in the THP-1 monocyte cell line [130]. *MALAT1* also regulates expression of the neighboring serum amyloid A3 gene in a mouse model [131]; serum amyloid A3 can activate MD-2-dependent p38 and NF-κB signaling [132]. These effects on inflammatory mediators suggest a potential mechanism through which *MALAT1* may contribute to ITP pathogenesis.

A pediatric case–control study assessed serum *MALAT1* and *THRIL* by quantitative PCR in 90 children with ITP and 90 healthy controls. Both transcripts were higher in patients, and their expression levels were correlated (r = 0.745). The cohort included newly diagnosed, persistent and chronic cases, and treatment exposure was associated with expression, complicating causal interpretation. The comparison with healthy individuals alone does not establish discrimination from other pediatric thrombocytopenias. The original article has a published erratum; any reuse of individual assay performance estimates should be checked against that correction and the full text [98].

The interpretation of the pediatric association should remain separate from the nuclear mechanisms described in Section 4.10. Relative serum expression can reflect the amount and persistence of released RNA, while nuclear *MALAT1* activity depends on its localization and partners within living cells. Longitudinal sampling before and after treatment could clarify whether *MALAT1* follows disease activity or treatment exposure; paired cellular measurements would be needed to connect such a trajectory to altered RNA processing. Any threshold intended for diagnosis would also require validation against other childhood causes of thrombocytopenia [98,99].

### 6.7. IFNG-AS1

*IFNG-AS1*, also known as *TMEVPG1* or *NeST*, is a long non-coding RNA located near the *IFNG* locus and participates in the regulation of IFN-γ expression. Its involvement in autoimmune diseases has been reviewed, including disease- and cell-specific differences in expression [81]. IFN-γ and Th1-related responses are implicated in ITP [133,134]. Importantly, *IFNG-AS1* findings differ between adult PBMC studies and childhood serum studies and should not be treated as interchangeable evidence [135,136].

Li and colleagues reported significantly lower *TMEVPG1*/*IFNG-AS1* expression in PBMCs from patients with active primary ITP than in healthy controls [135]. In vitro, expression of *TMEVPG1* and *IFNG* changed with stimulation duration: both increased after short stimulation, whereas *TMEVPG1* decreased and *IFNG* increased after 24 h. The authors proposed a possible feedback-regulatory role. In contrast, Ali and colleagues found increased serum *IFNG-AS1* and *GAS5* expression in children with ITP, with higher levels in persistent or chronic disease and in patients with partial or absent treatment responses [136]. Differences in age, disease phase, sample type and treatment may contribute to these contrasting observations.

Earlier experimental work linked *Tmevpg1* to IFN-γ regulation [137], while subsequent studies demonstrated that the magnitude of IFN-γ responses depends on both *Ifng-as1* transcription and local DNA architecture [82]. Together, these findings support investigation of *IFNG-AS1* regulation but do not establish that increasing its expression is an effective treatment for ITP.

*IFNG-AS1* illustrates why genomic position and the direction of a clinical association should not be conflated. It is a regulatory transcript at the *IFNG* region, but its alternative name does not by itself establish a simple antisense RNA–RNA duplex mechanism with *IFNG* mRNA. Experiments at the locus demonstrated contributions from local DNA organization as well as transcription. The opposite directions reported in adult PBMCs and childhood serum should therefore motivate compartment-specific studies, rather than a general conclusion that the RNA must always be increased or always decreased to support IFN-γ-dependent responses [82,135,136].

### 6.8. THRIL

*THRIL*, an immunoregulatory lncRNA associated with TNF-α and heterogeneous nuclear ribonucleoprotein L (hnRNPL), regulates TNF-α expression and thereby modulates innate immunity. TNF-α is the major pro-inflammatory and immunoregulatory cytokine that acts through TNFR1 and TNFR2 receptors, which are found on most cells. It promotes leukocyte recruitment, stimulates the production of other cytokines, and amplifies acute-phase responses, while also influencing immune tolerance [138].

Following TLR2 signaling in THP-1 macrophages, *THRIL* binds to hnRNPL to form a complex that associates with the *TNF* gene promoter and induces transcription. Conversely, reduced *THRIL* expression decreases TNF-α expression in macrophages [103]. These findings suggest a link between *THRIL* and autoimmune processes [139].

A study conducted at the Pediatric Clinic of Fayoum University Hospital in Egypt examined *THRIL* alongside *MALAT1* [98]. *THRIL* expression was significantly higher in patients with ITP than in controls. First-line glucocorticoid treatment reduced the expression of both *MALAT1* and *THRIL*.

Li and colleagues further demonstrated that *THRIL* regulates inflammatory responses through its interaction with hnRNPL and its effects on *TNF* transcription. It also influences the expression of CXCL10, CCL1, CSF1, and IL-8 [103]. These experimental effects provide biological plausibility for the circulating associations reported in ITP, but a causal *THRIL*–TNF-α pathway in ITP has not been established [98,103,105].

*THRIL* provides an RNA–protein mechanism complementary to the STAT3-linked pathways. Its association with hnRNPL and the *TNF* promoter was established in a macrophage-like experimental system, while its ITP studies measured circulating expression. Increased serum or plasma *THRIL* is therefore compatible with inflammatory activation but does not identify the producing cell or prove enhanced platelet phagocytosis. Connecting these observations would require matched cellular and circulating measurements, assessment of *TNF* transcription and protein production, and functional testing of platelet-directed effector responses. Such work would also clarify whether *THRIL* adds information beyond general inflammatory markers [98,103,105].

## 7. Role of LncRNAs in the Diagnosis and Treatment of ITP

Li and colleagues reported lower *TMEVPG1*/*IFNG-AS1* expression in patients with active primary ITP than in healthy controls [135]. A separate microarray study by Li and colleagues identified 1177 upregulated and 632 downregulated lncRNAs in primary ITP [6]. Fan and colleagues analyzed datasets GSE43177 and GSE43178 and identified 468 upregulated and 272 downregulated mRNAs, 134 upregulated and 23 downregulated lncRNAs, and 29 upregulated and 39 downregulated miRNAs. Their ceRNA-network analysis implicated pathways including cell adhesion, proteasomal degradation and mRNA degradation [78]. These exploratory expression signatures require independent validation before diagnostic use. The broader role of miRNAs in ITP has also been reviewed [79].

Persistent thrombocytopenia or failure to respond to treatment should prompt reassessment of the diagnosis and consideration of alternative causes, including myelodysplastic syndromes, drug-induced thrombocytopenia, bone marrow failure and inherited thrombocytopenia. Definitions of refractory or treatment-resistant ITP vary, and assessment should consider bleeding, platelet count, treatment history and patient-specific factors [7,8]. These challenges underscore the need for validated predictors of disease course and treatment response.

A study at Qilu Hospital of Shandong University in Jinan, China, included 102 participants: 41 adults with primary ITP, 20 patients with hematologic malignancies who had received chemotherapy, and 41 healthy controls. Exclusion criteria included autoimmune, respiratory, cardiovascular, and infectious diseases [105]. The mean age of patients with ITP was 40 years. The study compared *lnc-DC* and *THRIL* expression across the three groups. First described by Wang and colleagues, *lnc-DC* is strongly expressed in the cytoplasm of dendritic cells and cells of the monocyte lineage. It binds directly to STAT3 and promotes phosphorylation at tyrosine 705, supporting dendritic-cell differentiation [77]. *THRIL* regulates TNF-α expression at the transcriptional level. These observations motivate mechanistic investigation; circulating expression alone does not establish that either pathway causes ITP.

Jiang and colleagues found significantly lower plasma *lnc-DC* expression in adults with primary ITP than in healthy controls (*p* = 0.012) and patients with post-chemotherapy thrombocytopenia (*p* = 0.035) [105]. In contrast, Chen and colleagues reported elevated plasma *lnc-DC* in primary Sjögren’s syndrome, with particularly high expression in patients with associated ITP [140]. These findings arose from separate cohorts; they suggest context-dependent expression but do not establish the diagnostic accuracy of *lnc-DC* for directly distinguishing primary from secondary ITP.

In the adult study by Jiang and colleagues, plasma *THRIL* expression was higher in primary ITP than in healthy controls (*p* = 0.0005) and post-chemotherapy thrombocytopenia (*p* < 0.0001). The combined *lnc-DC*/*THRIL* model had an area under the ROC curve of 0.869 (95% confidence interval, 0.795–0.943) for distinguishing ITP from healthy controls [105]. This finding supports further evaluation of these markers rather than routine diagnostic implementation.

Between January 2017 and June 2018, a study at Soochow University Hospital in China enrolled 20 adults with ITP (13 women, 7 men) and a sex- and age-matched control group [76]. The study examined *GAS5* expression in PBMCs and its role in Th17-cell differentiation to identify potential therapeutic targets for ITP. Flow cytometry revealed a higher proportion of Th17 cells and a lower proportion of Tregs in patients with ITP than in controls. Th17 cells primarily produce IL-17. PBMCs from patients with ITP also showed lower *GAS5* expression and higher STAT3 levels [76]. Experimental *GAS5* overexpression promoted STAT3 ubiquitination and inhibited Th17 differentiation, without affecting Treg differentiation. *GAS5* also alleviated disease in an experimental mouse model; these findings support further preclinical investigation and do not establish therapeutic efficacy in patients [76].

*MEG3*, implicated in cancer biology, also shows increased expression in CD4+ T cells from patients with ITP compared with healthy controls [102]. *MEG3* participates in miRNA regulation, consistent with the broader role of lncRNAs in regulating miRNA expression [53]. Li and colleagues demonstrated that *MEG3* disrupts the Treg/Th17 balance by inhibiting miR-125a-5p expression [102], supporting its involvement in ITP pathogenesis. As discussed in Section 6.4, serum *MEG3* levels were significantly higher in patients with ITP than in healthy controls [124].

In the study by Naguib and colleagues, serum *MEG3* showed exploratory associations with organ bleeding, glucocorticoid response and subsequent splenectomy. The corresponding ROC areas were 0.805 (95% CI 0.569–0.945) (*p*  =  0.024) with a cutoff point of 5.1 and 100% sensitivity but 70% specificity, 0.853 (*p*  ≤  0.001) with a cutoff point of 7.2 and 94% sensitivity but 64% specificity and 0.750 (95% CI: 0.599–0.867) (*p*  =  0.049) with a cutoff point of 5.7 and 100% sensitivity but 51.28% specificity, respectively [124]. Receiver operating characteristic curve analysis was performed using Stata 16 to evaluate the predictive value of serum *MEG3* levels for bleeding severity and response to glucocorticoid therapy. Statistical significance was defined as *p* ≤ 0.05. Higher *MEG3* levels were associated with a greater risk of severe bleeding, whereas patients with sustained responses to glucocorticoid therapy had significantly lower levels than non-responders [124]. These results require external validation and do not justify an earlier splenectomy solely on the basis of *MEG3* expression.

lncRNA expression analysis is an investigational approach to ITP diagnosis and monitoring. Studies use different biological samples, including serum, plasma, whole blood, and isolated immune cells, which limits direct comparison of expression results (Table 1).

In an adult plasma study, Jiang and colleagues included 41 patients with ITP, 41 healthy controls and 20 patients with chemotherapy-associated myelosuppression. Plasma *lnc-DC* was lower and *THRIL* higher in ITP; the reported combined receiver operating characteristic area under the curve was 0.869 (95% CI 0.795–0.943). The authors reported 78.05% sensitivity for *lnc-DC* and 97.56% specificity for *THRIL* under their study conditions. These estimates arise from a small exploratory sample and should not be treated as externally validated clinical accuracy or transferred directly to a population with a different disease prevalence or differential diagnosis [105].

Cross-study comparison requires particular care. *MEG3* measurements in purified CD4+ T cells, *GAS5* in mixed mononuclear cells, *MALAT1*/*THRIL* in pediatric serum and *lnc-DC*/*THRIL* in adult plasma represent different biological matrices and patient groups. Differences in cell composition, treatment, hemolysis and preanalytical handling can produce apparent changes in circulating transcript concentrations. Prospective designs should standardize blood collection, processing and RNA normalization and document treatment timing, assess reproducibility and use independent validation cohorts with clinically relevant non-ITP thrombocytopenic controls. Candidate panels should be compared with routine clinical assessment and platelet count, with prespecified thresholds and confidence intervals [76,98,102,105].

Mechanistic confirmation requires more than co-expression or bioinformatic pathway prediction. For a proposed RNA–protein interaction, orthogonal binding assays, localization studies and loss-of-function and rescue experiments can test whether the interaction is necessary for the phenotype. For a proposed lncRNA–miRNA–mRNA network, target-site disruption and measurement of transcript abundance and subcellular co-localization are needed to assess plausibility. RNA-directed depletion and independent genetic strategies should be compared to distinguish effects of the mature RNA from changes in transcription or local DNA regulatory elements. Network analyses in ITP are hypothesis-generating until tested experimentally and reproduced [53,78].

Analytical validation should follow current quantitative PCR reporting recommendations, including MIQE 2.0. For lncRNA assays, this entails specifying transcript accession and isoform coverage, primer sequences, reverse-transcription strategy, amplification efficiency, detection limits and normalization. No-reverse-transcriptase controls are important where genomic DNA can generate a signal, and reference transcripts should be validated in the actual specimen and treatment setting. Reporting only fold changes without assay performance or uncertainty makes it difficult to compare cohorts or transport proposed diagnostic thresholds between laboratories [141].

## 8. Discussion

Primary ITP is a rare autoimmune disease, with an estimated annual incidence in adults of 3.3 cases per 100,000 individuals [1]. Loss of immune tolerance and autoreactivity against platelets are central to its pathogenesis, although the mechanisms underlying thrombocytopenia remain incompletely understood and are likely multifactorial. Although severe hemorrhagic complications are rare, identifying patients at risk is a major clinical challenge that informs treatment. Neunert and colleagues reported severe bleeding in 9.5% of adults and 20.2% of children with ITP [26]. Critical bleeding carries a risk of death or disability and includes intracranial, intraspinal, intraocular, pericardial, or retroperitoneal hemorrhage or intramuscular hemorrhage with compartment syndrome. Ongoing bleeding that causes hemodynamic instability or respiratory compromise also meets this definition [142]. Among patients treated for ITP in the United States between 2007 and 2016, intracranial hemorrhage occurred in 1.1% of adults and 0.7% in children and was associated with a mortality rate of 26.7% [143].

The studies reviewed suggest that lncRNA expression may help identify patients at increased risk of hemorrhagic complications. Liu and colleagues found that low *HCP5* expression was associated with bleeding severity and showed an independent association with a composite poor-prognosis outcome within a single cohort of 40 adults with ITP [5]. External validation and assessment of model stability are required before this can be considered a prognostic test. In children, Ali and colleagues reported increased serum *IFNG-AS1* and *GAS5* expression, with higher levels associated with persistent or chronic disease and partial or absent treatment responses [136]. The prognostic relevance of serum *IFNG-AS1* for bleeding remains to be established.

Naguib and colleagues reported higher serum *MEG3* levels in patients with organ bleeding and an association with platelet transfusion requirements [124]. Jiang and colleagues identified plasma *THRIL* and *lnc-DC* as candidate diagnostic markers in adults with primary ITP [105]. Further studies are needed to validate whether these markers improve bleeding-risk prediction beyond clinical assessment and platelet count.

Distinguishing primary ITP from secondary immune-mediated thrombocytopenia remains an important diagnostic challenge. Jiang and colleagues reported lower plasma *lnc-DC* expression in adults with primary ITP than in healthy controls [105], whereas Chen and colleagues found higher expression in primary Sjögren’s syndrome, particularly with associated ITP [140]. Because these were separate studies rather than a direct comparative validation cohort, the ability of *lnc-DC* to distinguish primary from secondary ITP remains unproven.

Glucocorticoids remain the most commonly used first-line treatment for ITP, but only approximately 50% of patients maintain a response at 6 months [39]. Identifying markers of glucocorticoid responsiveness could therefore help guide treatment selection. Lower *MEG3* levels have been associated with sustained responses to glucocorticoid therapy, suggesting potential predictive value, but it should be remembered that the effectiveness of this treatment is limited [124].

Splenectomy remains a treatment option for selected patients with ITP after consideration of medical alternatives, disease duration, bleeding, patient preference and infection risk [7,8]. Naguib and colleagues reported an association between serum *MEG3* and subsequent splenectomy in a small cohort [124]. This exploratory observation does not establish *MEG3*-guided indications or timing for surgery.

Studies linking altered lncRNA expression to ITP pathogenesis support investigation of therapeutic targets. *GAS5* has experimental support from cell and mouse studies [76]. *IFNG-AS1* is a candidate for further mechanistic research, but differences between adult PBMC and childhood serum findings must be resolved before therapeutic manipulation can be proposed [135,136].

The evidence base is limited by small, heterogeneous cohorts and a mixture of clinical associations, cell experiments, an animal model, and exploratory profiling. Among the clinical studies with reported sample sizes, ITP groups generally comprised 20–90 patients; the *GAS5* susceptibility study was larger, with 302 patients and 300 controls, but a case–control genetic association does not validate a diagnostic or treatment-response assay [127]. Several transcripts were measured in the same cohorts, so findings for different RNAs should not be counted as independent patient populations. Differences in age, specimen type, disease duration, and treatment exposure limit cross-study comparisons. Healthy-control designs may overestimate discrimination in clinical practice, and an ROC area does not establish calibration, incremental value, or improved outcomes. No pooled effect estimate or formal risk-of-bias score was calculated. The focused narrative selection, English-language restriction, and absence of a reproducible original search log create additional selection and reporting limitations; completeness cannot be claimed [10].

Taken together, these findings support potential clinical implications and further translational research. They do not establish an lncRNA test for distinguishing primary from secondary ITP, choosing splenectomy, or selecting an RNA-directed treatment. Future work should separate mechanistic causality from biomarker association and assess independent replication, assay reproducibility, clinically relevant comparators, and added value over routine assessment.

## Figures and Tables

**Table 1:** lncRNA expression in ITP varies according to the patient population and biological sample.

Long Non-Coding RNA	Expression Compared with Healthy Controls	Biological Sample	Patients (*n*)	Control (*n*)
*HCP5* [5]	Decreased in adults with ITP	Peripheral venous whole blood	40	40
*NEAT1* [101]	Increased in children with ITP	Serum	60	60
*lnc-DC* [101]	Increased in children with non-chronic ITP	Serum	60	60
*lnc-DC* [105]	Decreased in adults with primary ITP	Plasma	41	41
*MEG3* [102]	Increased in patients with ITP	Isolated CD4+ T cells	30	30
*MEG3* [124]	Increased in adults with ITP	Serum	45	45
*GAS5* [76]	Decreased in adults with ITP	Peripheral blood mononuclear cells (PBMCs)	20	20
*GAS5* [127]	Decreased in adults with ITP	Serum	302	300
*GAS5* [136]	Increased in children with ITP	Serum	88	88
*MALAT1* [98]	Increased in children with ITP	Serum	90	90
*IFNG-AS1*/*TMEVPG1* [135]	Decreased in adults with active primary ITP	PBMCs	54	45
*IFNG-AS1* [136]	Increased in children with ITP	Serum	88	88
*THRIL* [98]	Increased in children with ITP	Serum	90	90
*THRIL* [105]	Increased in adults with primary ITP	Plasma	41	41

## Data Availability

No new data were created or analyzed in this study. Data sharing is not applicable to this article.

## Abbreviations

ATXN2L	ataxin-2-like protein
BTK	Bruton tyrosine kinase
CCL	C-C motif chemokine ligand
ceRNA	competing endogenous RNA
circRNA	circular RNA
COVID-19	coronavirus disease 2019
CSF1	colony-stimulating factor 1
CXCL	C-X-C motif chemokine ligand
DC	dendritic cell
FLS	fibroblast-like synoviocyte
*GAS5*	growth arrest-specific transcript 5
*HCP5*	HLA complex P5
HCV	hepatitis C virus
HIV-1	human immunodeficiency virus type 1
hnRNPL	heterogeneous nuclear ribonucleoprotein L
IFN-γ	interferon gamma
*IFNG-AS1*	*IFNG* antisense RNA 1
IL	interleukin
ITP	immune thrombocytopenia
IVIg	intravenous immunoglobulin
JAK2	Janus kinase 2
*lnc-DC*	long non-coding RNA in dendritic cells
lncRNA	long non-coding RNA
*MALAT1*	metastasis-associated lung adenocarcinoma transcript 1
*MEG3*	maternally expressed gene 3
MHC	major histocompatibility complex
miRNA	microRNA
mRNA	messenger RNA
mTOR	mammalian target of rapamycin
*NEAT1*	nuclear paraspeckle assembly transcript 1
NF-κB	nuclear factor kappa B
NK	natural killer
PBMC	peripheral blood mononuclear cell
RA	rheumatoid arthritis
ROC	receiver operating characteristic
SIRT1	sirtuin 1
SLE	systemic lupus erythematosus
STAT	signal transducer and activator of transcription
Syk	spleen tyrosine kinase
TGF-β	transforming growth factor beta
Th	T helper
*THRIL*	TNF and hnRNPL-related immunoregulatory lncRNA
TLR2	Toll-like receptor 2
*TMEVPG1*	Theiler’s murine encephalomyelitis virus persistence candidate gene 1
TNF-α	tumor necrosis factor alpha
TNFR	tumor necrosis factor receptor
TPO-RA	thrombopoietin receptor agonist
Treg	regulatory T cell
